# Should I stay or should I go? Movement of adult *Triatoma sordida* within the peridomestic area of a typical Brazilian Cerrado rural household

**DOI:** 10.1186/s13071-017-2560-3

**Published:** 2018-01-05

**Authors:** Edson Santos Dantas, Rodrigo Gurgel-Gonçalves, Daniel Antunes Maciel Villela, Fernando Araújo Monteiro, Rafael Maciel-de-Freitas

**Affiliations:** 1 Laboratório de Transmissão de Hematozoários, Instituto Oswaldo Cruz, Fundação Oswaldo Cruz (IOC/ FIOCRUZ), Rio de Janeiro, Brazil; 2 Laboratório de Epidemiologia e Sistemática Molecular, Instituto Oswaldo Cruz, Fundação Oswaldo Cruz (IOC/ FIOCRUZ), Rio de Janeiro, Brazil; 3 Laboratório de Parasitologia Médica e Biologia de Vetores, Área de Patologia, Faculdade de Medicina, Universidade de Brasília, Campus Universitário Darcy Ribeiro, Brasília, DF Brazil; 4 Programa de Computação Científica, Fundação Oswaldo Cruz (PROCC/FIOCRUZ), Rio de Janeiro, Brazil

**Keywords:** Chagas disease, Movement, Dispersal, Vectorial capacity, *Triatoma*, Reduviidae, *Trypanosoma cruzi*

## Abstract

**Background:**

Chagas disease, or American trypanosomiasis, is an important neglected tropical illness caused by the flagellate protozoan *Trypanosoma cruzi*, which is primarily transmitted to humans by hematophagous insects of the subfamily Triatominae. Although knowledge on triatomine movement capabilities at the micro-geographical scale is of fundamental importance concerning the development of effective vector control strategies, it remains a poorly understood subject. Furthermore, survival rates and size estimates of natural populations are important topics to consider when evaluating transmission intensity.

**Results:**

The movement of adult *Triatoma sordida* within the peridomestic area of a rural Brazilian household was evaluated via mark-release-recapture assays. A total of 210 insects had their pronota marked with fluorescent dyes and were released at different distances from the chicken coop (two, five, ten and 20 m), and from the horse corral (27, 32, 35, 46 and 56 m). Recaptures occurred in three consecutive 15-day intervals. Specimens were successfully recaptured at all distances up to 32 m. Bayesian models were used to estimate recapture probability, survival rates (males vs females) and population size. Although recapture probability was inversely proportional to distance for both sexes, females were more affected by increased distance. On the other hand, no significant difference was detected in the survival rates between males and females in a 15-day period. Fisher-Ford and Bayesian models gave more accurate population size estimates than Lincoln method.

**Conclusions:**

*Triatoma sordida* adults were able to cover a distance of 32 m in 45 days. Recapture data modelling reveals that male dispersal was more effective suggesting that *T. sordida* males are more likely to contribute as potential colonizers of the peridomestic environment. Increasing the distance between the peridomestic structures and the sylvatic environment as much as possible appears to be a simple and feasible recommendation to reduce the contact rate between humans and infected bugs and ultimately Chagas disease transmission.

## Background

Chagas disease, or American trypanosomiasis, is an important neglected tropical disease caused by the flagellate protozoan *Trypanosoma cruzi*. This pathogen is most commonly transmitted to humans via the contact of infected faeces of hematophagous insects of the subfamily Triatominae (Hemiptera: Reduviidae) with the host’s mucous membranes [1, 2]. Other modes of transmission that have recently received attention include blood transfusion and ingestion of contaminated fruit juices [3, 4]. The former has led to the dissemination of the disease to developing countries where there is no transmission cycle, such as Spain and Japan [5, 6], and the latter has contributed to the generation of acute per os micro-epidemics in rural areas of northern Brazil [7].

*Triatoma sordida* (Stål, 1859) is the Chagas disease vector most frequently captured in the peridomestic environment in Brazil, particularly in areas where *Triatoma infestans* (Klug, 1834) has been eliminated by the Southern Cone Initiative [8, 9]. Knowledge of triatomine dispersal capabilities is key to the understanding of population dynamics and vector control. Although there is information available on the genetic structure and dispersal capabilities of *T. sordida* populations from Bolivia and Brazil at the macrogeographical level (inferred based on molecular markers, e.g. [10–13]), very little is known about small-scale triatomine dispersal (e.g. [14, 15]).

Information on triatomine dispersal capabilities at the micro-geographical level is of fundamental importance concerning the development of effective vector control strategies. The distance between the sylvatic and the domestic habitats seems to be a key factor that governs the success of the insect’s dispersive process [16, 17]. Surely, the ability of triatomine bugs to move (either by flight or simply crawling) within the peridomestic area and eventually invade and colonize human dwellings is of special interest [18].

Although survival rates and size estimates of natural populations are important topics to consider when evaluating transmission intensity, both have, to date, received little attention. The generation of reliable information on the abundance of triatomine populations is of great relevance as it provides estimates of recrudescent populations thus allowing for the evaluation of the effectiveness of vector control campaigns. The dispersal of *T. sordida*, for instance, has been studied in specific landscapes such as salt-flats (Salinas), or with the aid of experimental hen houses [19, 20]. The few estimates available suggest some individuals may present a long flight, with collection > 100 m from release point [19, 20].

The most effective technique for estimating insect dispersal capabilities, survival rates and population sizes, at the microgeographic scale, is the mark, release and recapture (MRR) method. It requires, however, that the long-lasting markers do not affect insect behaviour or survival, and also that released insects become randomly mixed within the local native population [21].

Because very little is known regarding the ability of anthropophilic bugs to disperse over small areas, we address this issue by focusing our investigation on the specific area referred to as the peridomicile. It is defined as the existing space situated in between the sylvatic and the domestic areas. The peridomicile is of particular epidemiological relevance as it is believed to serve as the liaison between sylvatic and domestic transmission cycles. Here, the dispersal capability of adult *T. sordida* within the peridomestic area of a rural Brazilian household was evaluated via mark-release-recapture assays. Furthermore, survival rates and size estimates of natural populations were analyzed.

## Methods

### Study area

**Fig. 1:**
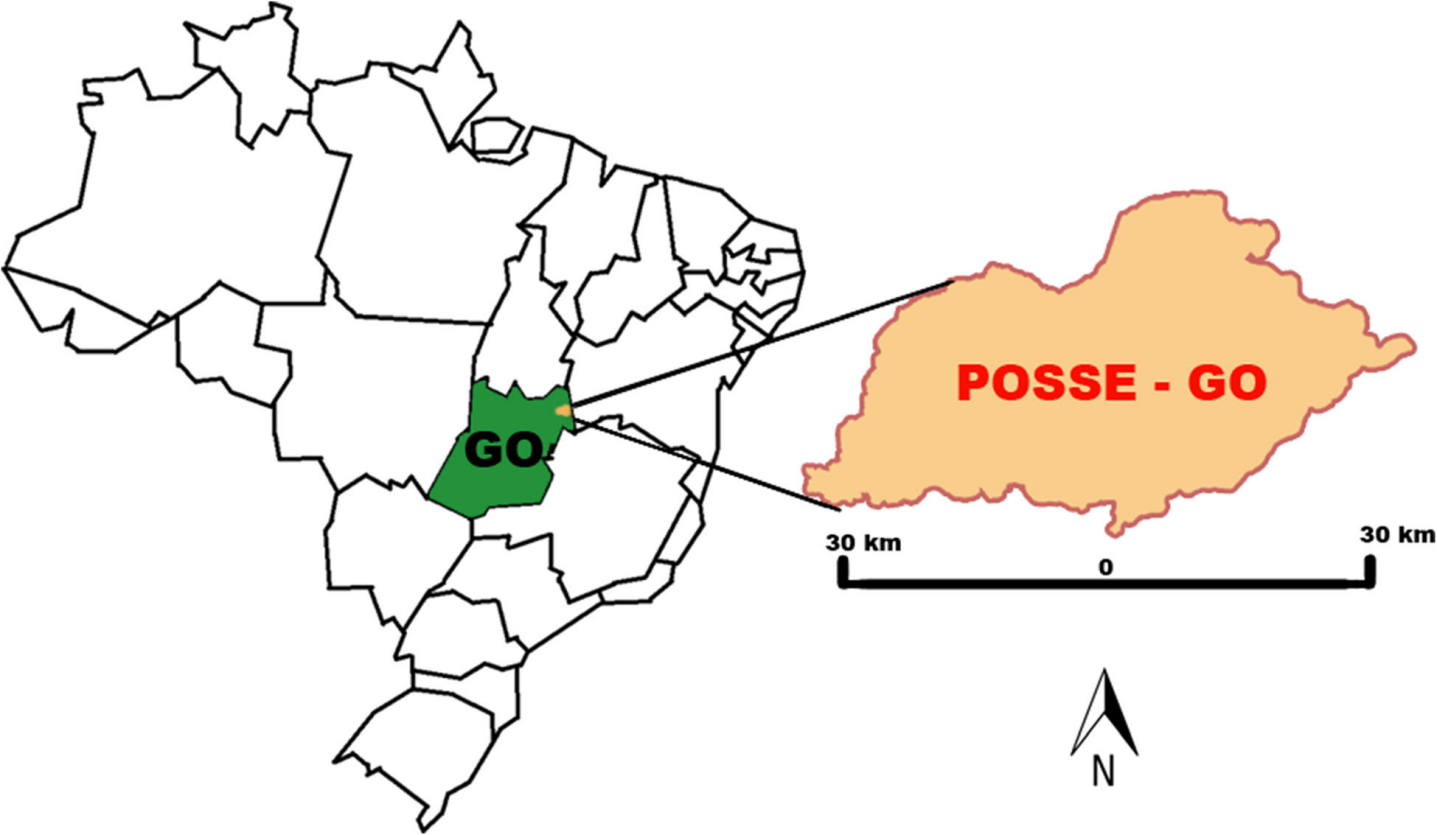
Location of the Municipality of Posse, Goiás State (GO), Central-West Region of Brazil

The study was conducted in one specific rural area in Posse, Goiás State, Brazil. This district is located at approximately 320 km from the capital, Brasília (Fig. 1). It encompasses an area of 2058.03 km^2^ and supports a population of approximately 35,000 inhabitants [22]. The vegetation is that of the Cerrado, a vast tropical plateaued savanna area that is the second largest Brazilian biome. It is a typical poor rural area with simple houses made of bricks and clay roof tiles. It is common to see peridomestic structures such as chicken coops (CC), horse corrals (HC), pigsties, and corn storage units in the region.

The household chosen for the experiment had two peridomestic annexes: a CC and an HC. The CC was 1.7 m high with an area of approximately 7.0 m^2^. Walls made with old timber were covered by an asbestos roof. The CC offered refuge to about eight chickens. A *T. sordida* colony was present. A rat nest was detected outside of one of the walls in an old tire with ragged cloth inside which was negative for the presence of triatomines. The HC was 3.5 m high with an area of approximately 90.0 m^2^ covered by a clay tile roof that provided shelter for two horses. Inside the HC there were small piles of timber where *T. sordida* specimens were found.

### *Triatoma sordida* field-collection and laboratory rearing

Triatomine collection was performed during five separate days from early April to late May of 2014 in 44 different rural properties in nine villages located on the surroundings of Posse district. The main objective of this field collections was to obtain enough insects to start a laboratory colony for further MRR experiments. Insects were captured manually with the aid of metal tweezers, from 9:00 am until 4:00 pm in peridomestic structures such as CC, HC, pigsties and corn storage units.

Collected specimens were transported to the Laboratory of Medical Parasitology and Vector Biology of the University of Brasília in 50 ml falcon tubes. At the laboratory, specimens were morphologically identified [23], placed in appropriate cages, and kept in the insectary (mean temperature of 30 ± 2 °C, with no humidity control) until a minimum of 210 adult insects (70 per release point) were obtained for the MRR experiments (i.e. adding the number of collected adults to fourth- and fifth-instars that molted into adulthood while in the insectary). Insects were fed on chickens every 15 days for 30–40 min.

### Mark, release and recapture (MRR) assays

Because very little is known regarding the capacity adult triatomines have in terms of small-scale dispersal (and our ability to recapture them), we adopted a simple strategy to assess insect movement that started by releasing marked specimens at a very close distance (2, 5 and 10 m) from its most likely destination point: the chicken coop. This assumption was based on historical data on triatomine bug surveillance conducted by local technicians and described in detail by Rossi et al. [24]. Evaluation of dispersal through longer distances was guaranteed by the presence of another manmade structure, the horse corral (HC), located farther away from the first three release points.

**Fig. 2:**
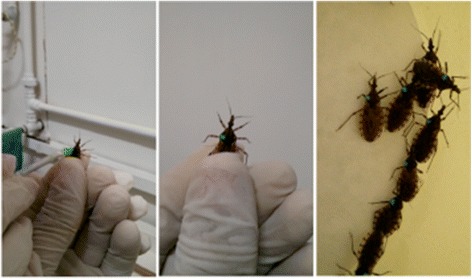
Process of painting the insect’s pronotum. Different colors were used to determine different points and dates of release

*Triatoma sordida* adults were marked with fluorescent dust (DayGlo Color Corp, Cleveland, USA) of different colors. Instead of using the dust dry, approximately 5 mg of each color was mixed in 2 ml of water to generate a thick liquid dye to be painted on the insect’s pronotum with the aid of a fine brush (Fig. 2). The use of fluorescent dust to mark insects is a common approach on MRR studies since the topic application does not seem to affect insect survival [25].

**Fig. 3:**
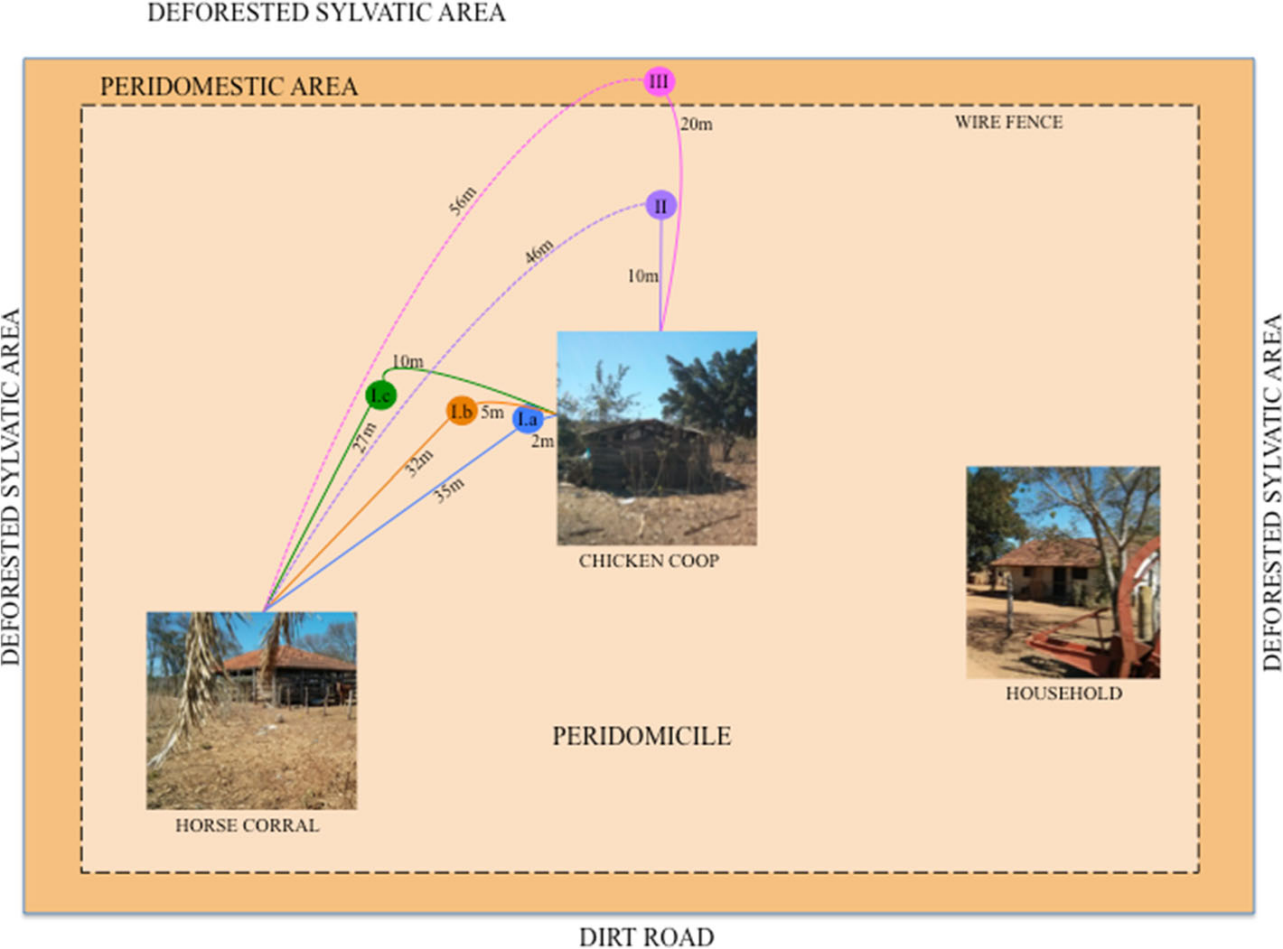
Area of study, release points, peridomicile structures and distances tested. I, first release event; II, second release event; III, third release event

A total of three marks, release and recapture (MRR) experiments were conducted to estimate triatomine movement, survival and abundance. In the first experiment, 210 adults were released from three points (70 from each) along with a straight line at the distances of 2 m (marked in blue), 5 m (orange), and 10 m (green) from the CC. These points were also 35, 32 and 27 m away respectively from the HC (Fig. 3).

The first recapture was conducted in the morning 15 days after the release and both structures (CC and HC) were inspected for the presence of marked *T. sordida.* The inspection was carried out by three health agents during 60 min in each recapture site. Both marked and newly captured (unmarked) adults received a purple dot on the pronotum and were released at dusk along a second transect 10 m away from CC and 46 m from HC. This was done to increase the release distances from the two peridomestic structures while remaining inside the peridomicile (Fig. 3).

Thirty days post-release we performed the second recapture event. Captured insects included specimens from the first and second releases, as well as new unmarked adults. All specimens received a pink mark on the pronotum and were released on the evening of the same day along with a third transect 20 m away from CC and 56 m from HC. The third and last recapture event were performed 45 days after the first release. By using different colors to mark insects, we were able to determine the exact day of release and distance travelled for every recaptured individual. All collected bugs were examined under UV light to determine whether there was a marker and if positive which color.

### Statistical analysis

We describe the capturing process as a Bayesian model. Each capture is described by a sequence of events, an MRR history, depending on the release times and capture occasion. The capture site is also part of the capturing history as a local variable *l* (dichotomous variable: HC, CC). The capture probability is described by *p*_*i,j*_ of history *i* and sex *j* (female/male). We model *p*_*i,j*_ such that log(*p*_*i*, *j*_) = *β*_0, *j*_ + *β*_1, *j*_*l*_*i*_ + *β*_2, *j*_*d*_*i*_, where *d*_*i*_ is the distance from releasing point to the capture site for history *i*,*β*_*0,j*_ is an intercept*,* and *β*_*1,j*_ and *β*_*2,j*_ are coefficients associated with local variable and distance, respectively. The few histories involving multiple recaptures contain multiple distance values associated with distance from each recapture to its releasing point. The number of captures described by history *i* for sex *j* is defined by ${N}_{i,j}\sim Binomial\left({R}_{i,j},{p}_{i,j}{\varphi}^{n_i}\right)$, where *R*_*i,j*_ is the number of releases, *φ* is the probability of survival during a 15-day period, and *n*_*i*_ is the number of surviving intervals.

Four different Bayesian models were tested. The first and simplest model (M0) considers survival rates and distance to capture location only. The second model (ML) increases in complexity by adding information on the collection location (whether insects were captured in CC or HC). The third model (MS) replaces “collection location” information with that on the sex of recaptured specimens. Finally, the fourth model (MLS) incorporates all parameters mentioned above (i.e. distance, survival rates, capture location, and sex). Model MS had the lowest deviance information criterion (DIC) and was therefore used in three of the four analyses performed. The MSL model was also used to specifically evaluate the likelihood of insects reaching either the CC or the HC.

Our Bayesian models are described using BUGS language [26, 27]. We use these models to run Markov Chain Monte Carlo simulations (MCMC), and as a result, to obtain posterior distributions for *β* parameters. Such distributions permit us to obtain estimates for the *β* parameters given our observations in the field, which explains how capturing probability is affected as release distance increases and the capture site varies (HC/CC). We used prior distributions *β*_*2,j*_~Normal(0, 100), *β*_*1,j*_~ Normal(0, 100), exp.(*β*_*0,j*_) ~ Beta(2,2).

Abundance estimates were obtained with the MRR data generated herein using the deterministic models of Fisher-Ford and Lincoln [25, 28–30]. These estimates were compared with the ones obtained via the chosen Bayesian model (Model MS).

## Results

A total of 44 peridomestic structures were inspected on the outskirts of Posse, and 30 (68.2%) were positive for the presence of *T. sordida*. A total of 583 *T. sordida* were collected (368 adults, with 232 males and 136 females, 143 N5, 42 N4, 8 N3, 2 N2 and 20 N1), with an average of 19.4 specimens per positive premise.

Of the 210 marked specimens released at the first MRR event (day 1), 16 (7.6%) were recaptured on day 15. A total of ten insects (eight males and two females) were collected in CC, whereas 6 (one male with an orange mark and thus released at 5 m away from CC, and four males and one female with a green mark released 10 m away from CC) were found in HC (Table 1). Therefore, 37.5% of the marked individuals recaptured were collected at HC, which is 27–32 m away, instead of at the CC located at 5–10 m from the release points (Fig. 2, Table 1). Additionally, ten new (unmarked) specimens were collected at CC.

**Table 1:** Color identification and number of *T. sordida* adults used in three MRR experiments with respect to release distances from the chicken coop (CC) and horse corral (HC), performed within a 45 day period

Event	Mark^a^	Release	Distance/CC (m)	Distance/HC (m)	Male/female	Recapture	Marked male/female CC	Marked male/female HC	Unmarked male/female wild
First MRR	B	Day 1	2	35	40/30	Day 15	2/1	–	
	O	Day 1	5	32	40/30	Day 15	5/1	1/–	3/7
	G	Day 1	10	27	40/30	Day 15	1/–	4/1	
Second MRR	B	Day 1	2	35	–	Day 30	2/1	–	
	O	Day 1	5	32	–	Day 30	–/1	–	
	G	Day 1	10	27	–	Day 30	–	–	
	P	Day 15	10	46	3/7	Day 30	–	–	13/5
	B, Pu	Day 15	10	46	2/1	Day 30	–	–	
	O, Pu	Day 15	10	46	6/1	Day 30	1/–	–	
	G, Pu	Day 15	10	46	4/1	Day 30	–/1	–	
Third MRR	B	Day 1	2	35	–	Day 45	1/–	–	–
	O	Day 1	5	32	–	Day 45	–	–	–
	G	Day 1	10	27	–	Day 45	1/–	–	–
	P	Day 15	10	46	–	Day 45	–	–	–
	B, Pu	Day 15	10	46	–	Day 45	–	–	–
	O, Pu	Day 15	10	46	–	Day 45	–	–	–
	G, Pu	Day 15	10	46	–	Day 45	–	–	–
	Pi	Day 30	20	56	12/5	Day 45	2/–	–	–
	Pu, Pi	Day 30	20	56	–	Day 45		–	–
	B, Pi	Day 30	20	56	2/1	Day 45	–/1	–	–
	O, Pi	Day 30	20	56	–/1	Day 45	–	–	–
	G, Pi	Day 30	20	56	–	Day 45	–	–	–
	B, Pu, Pi	Day 30	20	56	–	Day 45	–	–	–
	O, Pu, Pi	Day 30	20	56	1/–	Day 45	–	–	–
	G, Pu, Pi	Day 30	20	56	–/1	Day 45	–	–	–

*B* blue, *O* orange, *G* green, *Pu* purple, *Pi* pink

^a^The abbreviations on the “Mark” column refer to the color insects were marked

In the second MRR event, 25 individuals received a purple mark (15 of them received the second mark, and 10 new specimens were marked for the first time) and were released on day 15 at 10 and 46 m away from CC and HC, respectively. On the morning of day 30, we recaptured six marked individuals, four of them (three males and one female) from the first release (day 1) and therefore captured for the first time; two recaptured for the second time, and 18 unmarked insects (Table 1). During the second recapture event, all triatomine bugs were recovered in CC.

In the third MRR event, 23 individuals (six already recaptured in the first and second MRR and 18 new specimens) received a pink mark and were released at 20/56 m away from CC/HC (Table 1). At this point, there were triatomine bugs in the field with one, two or three markers, which allowed us to determine the distance marked individuals were released and for how long they survived in the field. A total of five marked insects were recaptured on day 45, including two males from the first release (day 1), one released at 2 m (blue mark) and another at 10 m (green mark). The other three triatomines were marked with a pink dot and thus released at 20 m on the third MRR event (day 30). No new individuals were captured at this time.

**Fig. 4:**
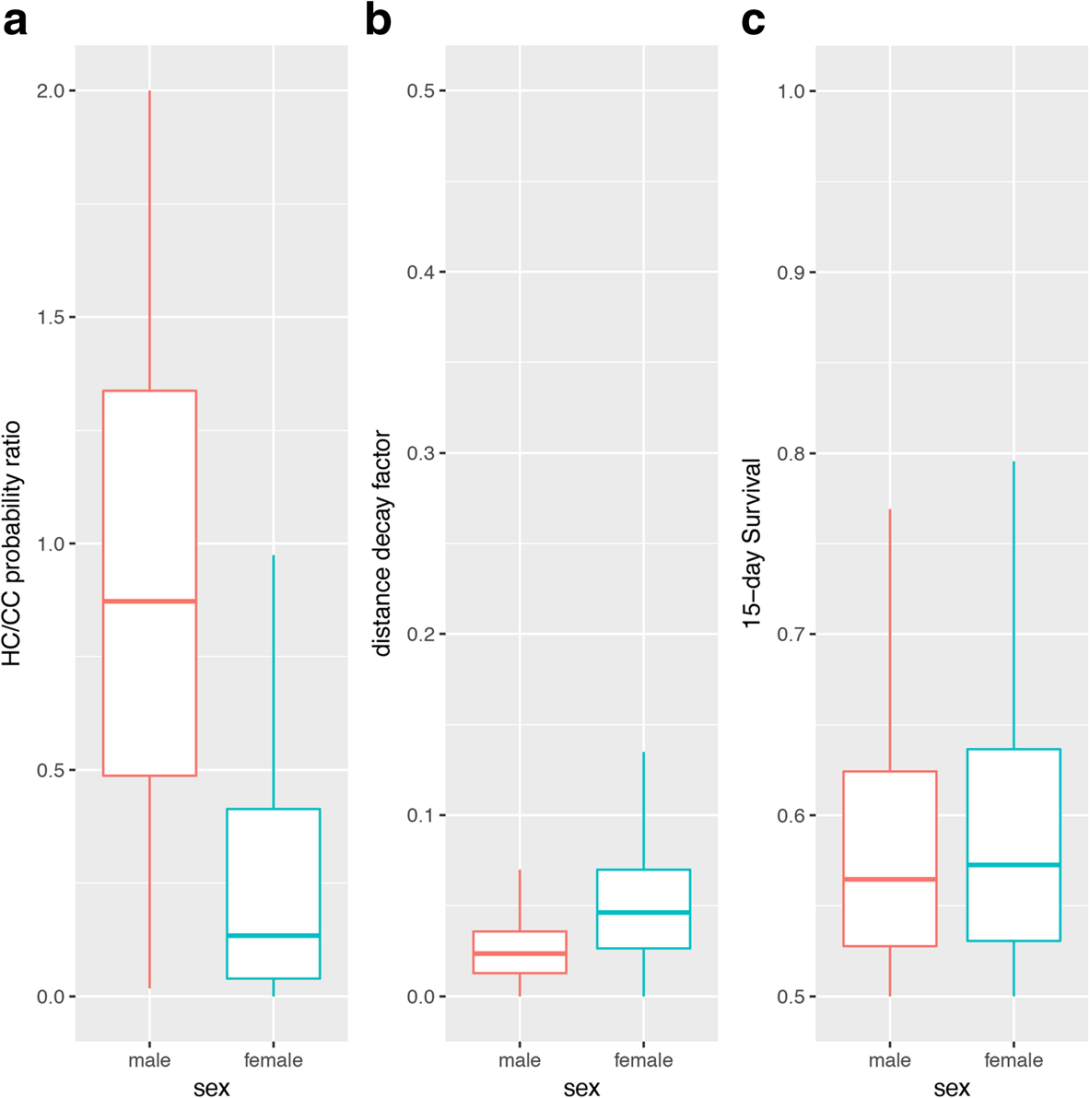
Bayesian models. **a** Capture probabilities for horse corral/chicken coop ratios for both males and females (Model MSL). **b** Effect of distance between release and recapture locations on the probability of *T. sordida* reaching either peridomestic structure (Model MS). **c** Male and female survival probabilities for three 15-day periods (Model MS)

Recapture likelihood seems to be sex-biased as females were found more often at CC than at HC. The ratio HC/CC for male recapture is close to 1 with a wide distribution, indicating that males, instead, appear not to have a particular preference for either peridomestic structure (Fig. 4a). Recapture probability showed an inversely proportional relationship to distance (from release to collection sites). Capture probability decreases by factors given in Fig. 4b for both males and females. This analysis shows that distance is a limiting factor for triatomine dispersal, especially for females. Although subject to a greater predation risk by moving longer distances to reach HC, no statistical difference was detected between male/female survival probabilities (Fig. 4c).

Based on the results seen in Fig. 4b, a graph was generated to depict the probabilities associated with the ability of *T. sordida* to reach both peridomestic structures within distances of up to 100 m. Nonetheless, there is still a low possibility of these insects reaching CC or HC even at greater distances.

**Fig. 5:**
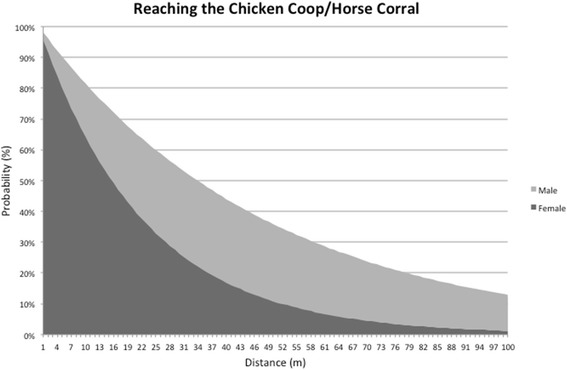
Probability of *Triatoma sordida* adults of reaching any of the two peridomestic structures, the chicken coop or the horse coral, for different distances

The probability of reaching a peridomestic structure when at a distance of, say, 20 m is much higher for males (approximately 60%), than for females (40%) (Fig. 5). This suggests *T. sordida* males are more likely to contribute as potential colonizers of the peridomestic environment.

Estimates based on the deterministic Fisher-Ford model were all within the credibility interval of the Bayesian method (Model MS). On the other hand, estimates for the Lincoln model lay outside the Bayesian credibility interval (e.g. the credibility interval during the second recapture pointed to 18–136 males, whereas the Lincoln estimate was 780 males; Table 2). A great rise in abundance estimates was observed between the first and second recapture events (Table 2). It seems unlikely that this discrepancy resulted from real population size increase, but rather could be due to the collection of previously uncaptured “resident” triatomines during the second recapture event.

**Table 2:** Relative abundance of *Triatoma sordida* based on the Bayesian method (Model MS) and the deterministic Fisher-Ford and Lincoln methods

Abundance estimate	Bayesian method			Fisher-Ford	Lincoln
Population/Occasion (first or second recapture)	Mean	Median	Credibility interval	Estimate	Estimate
Male/first recapture	29	24	10–73	32	105
Male/second recapture	53	45	18–136	70	780
Female/first recapture	12	9	4–41	40	135
Female/second recapture	21	15	6–69	40	450

## Discussion

The results presented here on the dispersal capabilities of adult *Triatoma sordida* specimens in the peridomicile of a rural central Brazil household were obtained using MRR. With the development of an efficient marking system that used long-lasting marks of different colors that could be systematically added to the pronotum of recaptured insects, we were able to generate relevant new information on the dispersal and dynamics of a peridomestic *T. sordida* population. This sort of information, i.e. triatomine dispersal capabilities on a micro-geographical scale, although of obvious epidemiological relevance, seems to have remained elusive to investigators throughout the years.

The most salient feature of this study was the determination that, in a 45-day interval, adult *T. sordida* specimens released in the peridomestic area of a typical rural house in central Brazil, are capable of actively dispersing across distances of up to 32 m. The cues defining the orientation of such dispersal are unclear as, contrary to the expectation that insects would be more prone to move towards the closer CC, some dispersed further and in a different direction to achieve the more distant HC. This is an important finding as it challenges the belief that *T. sordida* has a remarkable preference for avian hosts.

The establishment of distances, in a given period, within which insect colonization (or recolonization) are likely to occur is fundamental for the understanding of Chagas disease epidemiology and constitute a major step forward towards long-term sustainable vector control interventions.

The observation that there is an apparent discontinuity in the capture success, in a 45-day period, at the distance of 32 m is of special epidemiological interest (assuming no insects flew beyond the maximum distance of 56 m, see below). This finding is corroborated by the results of statistical models applied to the data obtained. The major focus of all multinational Chagas disease control initiatives launched to date has relied on the spraying of infested houses and peridomestic structures with residual insecticides [31]. However, sylvatic populations of triatomine bugs may migrate and re-colonize peridomestic structures soon after control activities [32–34].

The evaluation of *T. sordida* infestation in 406 rural households of Southeast Brazil led to the collection of 772 insects before insecticide spraying (98% in the peridomicile [35]). A similar number of insects were captured in the two collections performed after spraying (7 and 12 months afterwards). It was observed that 62.9% of captured peridomestic *T. sordida* was located near the sylvatic area (12–300 m). Authors attributed the finding of high numbers of specimens after spraying for local insect survival and immigration from sylvatic areas [35]. Although informative, the lack of utilization of a marking technique prevented the authors from drawing more objective conclusions based on the data obtained. In addition, it seems that *T. sordida*’s reduced dispersal capability would have precluded dispersal over larger areas. Therefore, it would be more plausible to suppose that insects collected after spraying constituted a recrudescent population composed of surviving individuals.

Direct MMR observation revealed 27 and 32 m as maximum travelled distance towards a peridomestic structure by females and males (one individual each), respectively. As expected, the probability of reaching either CC or HC is strongly dependent on distance. Artificial CCs placed directly in the sylvatic environment became readily infested by wild *T. sordida* populations [19]. Therefore, one practical initiative to reduce the contact rate between human hosts and triatomine bugs would be to construct peridomestic structures as far as possible from the sylvatic environment. Although our study only focused on the peridomestic area, it is reasonable to assume that there is where any new migrant will land if, for instance, it flies in from the sylvatic habitat. In this scenario, the peridomestic structures could act as “stepping-stones” towards any domestic structure. The greater the distance between these three areas (sylvatic environment, peridomestic structures, domestic structure), the lower the likelihood of domestic colonization. Following this line of reasoning, Fig. 5 shows, for example, that if positioned at least 55 m away, the probability of colonizing CC or HC drops to less than 30% for males and 10% for females.

Historical data regarding triatomine movement is based on a variety of marking methods, capturing techniques and data analysis, which compromise the direct comparison of results generated. In general, limited evidence has been produced with regard to the active dispersal capabilities of these insects, with most available data focusing on vector flight capacity. Gómez-Núñez [36] was one of the first to address the issue of triatomine micro-geographical dispersal and communication between sylvatic and domestic transmission cycles. By internally marking specimens of *Rhodnius prolixus* with gold-covered CO_60_ wire tags and tracking them with Geiger and scintillation counters, he observed the movement between palm trees to houses in rural Venezuela. During 40 days, *R. prolixus* dispersal was motivated mainly by starvation and did not exceed 15 m. Besides, migration seemed to be unidirectional, from palms towards houses [36]. A recent study [37] suggests through mathematical models that the closer the palm of the house, the greater is the chances of it being visited by *R. prolixus*.

Schofield et al. [20] carried out an MRR experiment with *T. sordida* adults in salt-flats (salinas) of Argentina aiming to determine flight dispersal behaviour. The majority of released insects were not recaptured, suggesting dispersal capability superior to 200 m on that specific landscape. Lehane and Schofield [38] performed MRR experiments with fluorescent marked *T. infestans* males and observed that bugs were able to disperse by active flight for more than 100 m in field sites from Brazil and Argentina. As in Schofield et al. [20], both experiments focused on flight initiation with use of a brick platform to help trigger take-off. The additional stimulus was provided by kerosene lamps as attraction points (only for the latter study).

Our study should not be directly compared with these important earlier investigations. They aimed at determining insect dispersal by flight in a scenario of “fleeing from an inhospitable environment (i.e. salt flats) with the aid of a launching platform”. Our results should rather be interpreted as “having escaped from a condition of hardship in the wild, such as the described above, and successfully landing in a peridomestic area, what do adult *T. sordida* do?”

Could the insects have dispersed (flown) further than the maximum distance of 56 m investigated here? Possibly, although this issue was beyond the scope of the present work. Nonetheless, available evidence suggests that in the absence of a launching platform adult *T. infestans* will not take-off spontaneously from the ground level as observed by the lack of adults to overcome a physical barrier and return into experimental huts [39]. Moreover, Forattini et al. [19] demonstrated that although wild *T. sordida* will readily invade and colonize experimental CCs in both deforested and pasture areas of the Cerrado, dispersal in the opposite direction was negligible: only one insect out of 172 flew back into the sylvatic area. This indicates that *T. sordida* will likely disperse toward food and shelter and not away from it [40].

We attempted to replicate the natural conditions seen in rural Cerrado areas as much as possible by choosing a specific site that had two natural sources of attraction for triatomines: a CC and an HC. Therefore, we believe that the *T. sordida* we released in this particular peridomicile most likely achieved the CC and HC crawling, since insects were monitored for 10 min after release on the ground at dusk and none started flight.

There is a large body of work on the estimation of abundance, survivorship and other important population descriptors for wild animals [41]. Many methods, however, face limitations when applied to the analysis of vector MRR data where specimens are individually marked and often recaptured multiple times [42]. The experimental design used here, notably the use of a new marker color for different release points and events allowed for important estimates such as (i) capture probability as a function of distance to either HC and CC, and (ii) triatomine survivorship, to be made.

The MRR method, as almost all experimental techniques, is not perfect and will present shortcomings in certain situations. In our case, one particular limitation was the low recapture rate (which was nonetheless still within the range seen for other vector groups such as mosquitoes, e.g. [42, 43]). Dispersal from a central point will lead inevitably to what is known as “dilution effect” where individuals will spread out over a progressively greater area and thus become more difficult to recapture. We attempted to circumvent such challenge by focusing our searches solely on the two peridomestic structures available, the CC and HC.

Knowledge of vector survival is key for the estimation of how long an infected vector may transmit a pathogen to a susceptible host. Our survival model showed that there is no difference in survival rates between males and females during a 15-day interval. Surprisingly, results indicate that *T. sordida* males disperse further and thus may colonize the peridomestic structures more effectively than females. Therefore, evidence suggests our paper is the first to demonstrate that sex per se can influence *T. sordida* vectorial capacity.

*Triatoma sordida* abundance estimates for the first two recapture events show that the number of adults of both sexes significantly increased in each 15-day period according to the Bayesian, Lincoln and Fisher-Ford methods (excluding Fisher-Ford estimates for females). This sudden increase in population size is unlikely to be real, but rather reflect the collection of previously uncaptured wild insects on the following recapture event. Among the methods used to estimate *T. sordida* population size, the Lincoln method gave discrepant results since they pointed to a 7.4-fold increase in male population size in a 15-day period. A possible explanation for the discrepancy of the Lincoln method is that survival probability was not considered, only numbers of released and recaptured individuals.

## Conclusions

The implementation of MRR to estimate dispersal capability of triatomine vectors can significantly improve vector control strategies by determining, for instance, the distance within which wild insects are more likely to colonize the peridomestic structures such as CC or HC. *Triatoma sordida* showed a dispersal capability, within 45 days, limited to 32 m from the release point, not being collected beyond this threshold. Therefore, increasing the distance as much as possible between the peridomestic structures and the sylvatic environment, as well as from the peridomestic structures and the domicile, seems to be a simple and feasible practice to reduce the contact rate between humans and infected bugs and, by corollary, Chagas disease transmission.

## Abbreviations

CC: Chicken coops
DIC: Deviance information criterion
GO: State of Goiás
HC: Horse corral
MCMC: Markov Chain Monte Carlo
MRR: Mark, release and recapture
